# Stress Perception, Sleep Quality and Work Engagement of German Outpatient Nurses during the COVID-19 Pandemic

**DOI:** 10.3390/ijerph19010313

**Published:** 2021-12-28

**Authors:** Monika Bernburg, Mara Shirin Hetzmann, Natascha Mojtahedzadeh, Felix Alexander Neumann, Matthias Augustin, Volker Harth, David Alexander Groneberg, Birgit-Christiane Zyriax, Stefanie Mache

**Affiliations:** 1 Institute of Occupational, Social and Environmental Medicine, Goethe-University Frankfurt am Main, 60590 Frankfurt, Germany; monika.bernburg@gmail.com (M.B.); groneberg@med.uni-frankfurt.de (D.A.G.); 2 Institute for Occupational Medicine and Maritime Medicine (ZfAM), University Medical Center Hamburg-Eppendorf (UKE), 20459 Hamburg, Germany; mache@med.uni-frankfurt.de (M.S.H.); n.mojtahedzadeh@uke.de (N.M.); harth@uke.de (V.H.); 3 Midwifery Science-Health Services Research and Prevention, Institute for Health Service Research in Dermatology and Nursing (IVDP), University Medical Center Hamburg-Eppendorf (UKE), Martinistr. 52, 20246 Hamburg, Germany; fe.neumann@uke.de (F.A.N.); b.zyriax@uke.de (B.-C.Z.); 4 Competence Center for Health Services Research in Vascular Diseases (CVvasc), Institute for Health Services Research in Dermatology and Nursing (IVDP), University Medical Center Hamburg-Eppendorf (UKE), 20246 Hamburg, Germany; m.augustin@uke.de

**Keywords:** outpatient care nursing, ambulatory care, stress, worries, work engagement, coronavirus, pandemic

## Abstract

Since the outbreak of the COVID-19 pandemic, outpatient nurses have been exposed to a double burden of already known occupational and new pandemic-related stressors. Recent studies suggest that increased pandemic-related stress can affect mental health and promote the development of negative mental health outcomes for nurses. This includes a decrease in sleep quality and work engagement. In addition, certain groups appear to be particularly vulnerable to pandemic-related stress. The aim of this study was to investigate the stress perception of German outpatient nurses during the COVID-19 pandemic. The aim was to determine associations between their pandemic-related stress and variables such as sleep quality, work engagement, pandemic-related worries and concerns. For this purpose, a questionnaire was developed based on well-established measurement instruments such as the 10-item Perceived Stress Scale, the Pittsburgh Sleep Quality Index and the Copenhagen Psychosocial Questionnaire to conduct a cross-sectional online survey among outpatient nurses from Germany. Participants (*n* = 166) showed rather moderate overall pandemic-related stress levels, good sleep quality, high work engagement, and moderate pandemic-related worries and concerns. Pandemic-related stress proved to be a predictor of decreased sleep quality and work engagement of outpatient nurses with weak effect sizes. Despite the surprisingly moderate stress levels, the effects of pandemic-related stress on selected aspects of participants’ mental health could be demonstrated. Therefore, behavioural and organisational health promotion measures are recommended to support outpatient nurses during the pandemic. However, further research is needed to determine the causal relationships and long-term effects of pandemic-related stress on the mental health of outpatient caregivers.

## 1. Introduction

Since the outbreak of COVID-19, nurses have been at the forefront of the fight against the virus, maintaining patient care against all odds. As a result, nurses face a double burden of already known occupational and new pandemic-related stressors. These circumstances have been shown to affect nurses’ stress experience and thus also their mental health [1]. Indeed, it is known from previous epidemics that perceived stress can be elevated during epidemic or pandemic states [2]. The identified COVID-19 related stressors that are experienced by nurses, so far, are multifactorial. Among others, pandemic-related worries and concerns, as well as a lack of pandemic-related information, could be identified as some of the main triggers for pandemic-related stress among nurses [3,4,5]. Likewise, the mental health outcomes of the pandemic-related stress experience of nurses in national and international contexts are already evident. In a very short time, the COVID-19 outbreak has been associated with an increase in nurses’ stress levels, which fosters the development of adverse mental health outcomes such as depressive symptomology, major depression, mental exhaustion, sleep disturbances, and adjustment and anxiety disorders [3,6,7,8,9]. In addition, some studies provide evidence that specific pandemic-related stressors had an even greater impact on outpatient care than on full or partial inpatient care, e.g., the risk for infection, shortages in personal protective equipment (PPE), and the implementation of occupational safety measures against an infection [10,11,12]. Moreover, the increased experience of stress during the COVID-19 pandemic is shown to harm nurses’ work engagement [13]. 

### 1.1. Current State of Research: Stress Experience, Sleep Quality and Work Engagement of Nurses during the COVID-19 Pandemic

Since the beginning of the COVID-19 pandemic, nurses have been at the forefront of the fight against the virus and are therefore particularly affected by the pandemic-related changes in working conditions and new occupational stressors. Current literature therefore suggests that mental health effects and mental health outcomes among nurses triggered by the pandemic-related changes in their daily work are of particular importance [1]. Currently, there is limited evidence on COVID-19-specific stressors and the associated related mental health outcomes in German outpatient nurses. In a recent study, German outpatient nurses described daily masking requirements, lack of PPE and tightened hygiene regulations as stressful during the pandemic. In addition, outpatient nurses perceived a higher workload and emotional demands due to the fear of contracting COVID-19 or infecting others [12]. Further study results should be considered. However, further evidence relates primarily to the inpatient care sector. Nevertheless, these findings can be further supplemented by research results from previous epidemics and pandemics.

As shown in reviews by Bohlken et al. [7], Mulfinger [14] and Schulze and Holmberg [15], numerous national and international studies confirm an increase in adverse mental health outcomes among nurses since the outbreak of COVID-19, regardless of the setting. In this context, there is evidence that multifactorial pandemic-related stress factors contribute to and exacerbate nurses’ experiences of stress. Identified psychological consequences of pandemic-related stress of varying severity include anxiety, sleep and adjustment disorders, as well as symptoms of mental exhaustion, depression, and burnout. Furthermore, Bohlken et al. [7] identified determinants of the severity of psychological symptomatology in healthcare professionals during the COVID-19 pandemic such as age, gender, occupational group, occupational specialisation, type of work, and contact to COVID-19 positive patients. In this respect, the female gender and younger employees were more susceptible to COVID-19 related stress. The fact that adverse mental health outcomes among nurses are increased in epidemic or pandemic states are consistent with findings in the review by Mulfinger et al. [14], who found an increase in mental stress and adverse mental health outcomes among healthcare professionals during epidemics such as SARS, MERS, and Ebola. Comparatively, their review also reflected that certain groups, such as female and young employees, were more affected by mental stress than others. Furthermore, several studies from the international context show that fewer years of work experience are a predictor for the enhanced stress experience of healthcare professionals during the COVID-19 pandemic [16,17,18].

In this context, the question arises as to which factors exactly contribute to the fact that the stress level of nurses increases in such states of emergency. Therefore, the actual state of caregiving prior to the outbreak of the COVID-19 pandemic should be considered. As mentioned earlier, the workload and work density of nurses have been increasing for years due to staff shortages, which has a significant impact on their stress levels [19]. During the course of the COVID-19 pandemic, this situation was exacerbated by increasing staff absences, mainly due to sickness-related absences or quarantine measures. For example, outpatient care facilities reported that up to 10% of the staff were temporarily absent. Furthermore, the additional workload was caused by the implementation of new necessary infection control and hygiene measures against COVID-19. As a result, outpatient nurses reported a further increase in their workload and work intensification. On average, the additional workload in outpatient care was estimated at 40 min per shift [10,12]. Analogously, Rothgang et al. were able to demonstrate similar conditions for nurses in retirement homes in their study [20]. Comparable findings were also collected in inpatient care, where changed working time models and work teams were additional stressors [3,5]. 

During periods of high workload and work-related stress, recovery and regeneration from work are especially important for maintaining mental health [21]. However, the staff shortages and increased workload led to temporary bans of taking leave during the COVID-19 pandemic in the in- and outpatient care setting [10,20]. Paradoxically, however, short-time work has also been reported in outpatient care despite increased workloads [10,22]. An imbalance between increased workload and insufficient rest may have various consequences for mental health. Work-related stress, especially due to staff shortages and high workload, has been shown to be associated with poor sleep quality in nurses [23]. Similarly, Salari et al. concluded in their review that the prevalence of sleep disorders among nurses and physicians has also increased since the COVID-19 outbreak, which was substantially caused by increased pandemic-related stress levels [24]. These previous findings could thus explain possible correlations between increased stress and unfavourable mental health outcomes such as sleep disturbances that have been observed among nurses since the COVID-19 outbreak [7,25]. On this basis, it could be assumed that pandemic-related stress had a psychological impact on outpatient nurses as well, which might have affected their sleep quality during the COVID-19 pandemic.

Moreover, the medical sector is considered a high-risk setting for the transmission of SARS-CoV-2, as medical or caregiving procedures with direct patient contact expose healthcare professionals to a high risk of infection [26]. In this context, high numbers of COVID-19 positive health professionals were reported worldwide, similar to those reported during the SARS pandemic of 2002 to 2003 [27,28]. In total, 140,000 confirmed COVID-19-positive cases among health professionals including outpatient care services were registered by the end of March 2021 [29]. The research literature also suggests that outpatient nurses are at higher risk of COVID-19 infection, especially if their clients have a history of infection. In fact, the risk of infection among outpatient nurses is even higher than among nurses working in retirement homes, which could be due to the closer physical contact with clients in outpatient care [10]. In this context, the already described cramped and poorly ventilated spatial conditions in some of the client residences probably also play a role in the comparatively higher risk of infection among outpatient nurses [30,31]. 

It must be emphasised that risk groups for a severe course of COVID-19 can also be found among healthcare professionals [32,33]. However, the high risk of infection is not only a physical risk but is also a psychological burden with far-reaching effects on the mental health of healthcare professionals. This is because the condition of a pandemic is associated with worries and anxieties in its consequences and through the risk of infection. In the study by Goulia et al. during the A/H1N1 pandemic in 2009, more than half of the participants (*n* = 469) reported worrying about the pandemic [34]. The most common concern was about infecting family and friends and the health consequences of the disease. [34]. Similarly, an Italian study from the first wave of the COVID-19 pandemic indicated that inpatient nurses perceived their risk of being infected with COVID-19 as very high and were mostly worried about the risk of infection with COVID-19 for their patients, family members, and friends. As a psychological outcome, sleep disturbances occurred in a majority of the study’s participants [35]. Likewise, a recent Japanese study among *n* = 4386 frontline and non-frontline healthcare professionals proved that nearly all of their participants were worried about the COVID-19 pandemic and found a high degree of worry and concerns in almost 80% of the participants. In this respect, there was no difference in pandemic-related worries and concerns between frontline and non-frontline healthcare professionals [36]. However, other studies provide evidence that stress levels are higher among healthcare professionals who have direct contact with COVID-19 positive patients than those without contact with infected patients [4,13,37]. Similar findings were also identified among nurses during the SARS outbreak, with those with direct contact with SARS-infected patients experiencing more severe psychological consequences such as anxiety, depression, burnout, somatization, post-traumatic stress disorder, than those without direct contact with infected patients. Interestingly, other studies also revealed that for some nurses, concern about transmitting the virus to patients and their families, as well as about the health of others, even outweighed fear of the consequences of a self-infection with SARS-CoV-2 [3,5,13,36,37]. All these findings are consistent with the conclusions from previous epidemics, where concern about the risk of infection, as well as concerns about possible consequences for one’s own health or family, was perceived by health professionals as the greatest psychological burden [14]. 

However, the literature suggests that it is not only the risk of infection that causes worries and concerns among nurses during the COVID-19 pandemic. In several studies infection prevention measures such as isolation or quarantine of COVID-19 positive patients led to strong concerns among nurses about the quality of patient care, e.g., due to the lack of physical contact in the intensive care setting [3,38,39]. Concerns about quality of care were also found among German outpatient nurses, as their clients used their care services significantly less often due to fear and uncertainty about the risk of infection posed by nurses [5,10,13]. In turn, the decline in turnover of outpatient care services also triggered existential and financial concerns among outpatient nurses [9,22,40,41,42].

Moreover, considering the JD-R model (Demerouti & Nachreiner, 2019), the effects of pandemic-related stress on nurses’ work engagement have also been observed. On the one hand, high levels of pandemic-related stress and worries about their health were found to be associated with significantly lower work engagement in nurses [43]. On the other hand, worries about the well-being of patients are associated with an increase in nurses’ work engagement [13]. These findings are consistent with the conclusions of a Chinese study during the first wave of the COVID-19 pandemic, which found that work engagement was negatively correlated with stress and workload among inpatient nurses [44]. Therefore, it might be plausible that a negative impact of pandemic-related stress also occurred among outpatient nurses as well, although no study results are available on this yet.

However, it would not be sufficiently differentiated to reduce the stress experienced by nurses during the COVID-19 pandemic exclusively to occupational stress. In fact, there is evidence that nurses also felt pandemic-related stress in their personal lives. Rheindorf et al. found that many nurses perceived their private lives as very restricted and cited the lack of respite from work due to public life restrictions as one of the greatest challenges to their mental wellbeing [9]. Again, female and younger respondents felt more stressed by the restrictions [11]. In addition, new stressors in family life emerged during the COVID- 19 pandemic, such as increased conflict, childcare, and home schooling [3,7].

### 1.2. Research Gap

International and national studies provided the first evidence of pandemic-related stress and related mental health outcomes among nurses since the outbreak of the COVID-19 pandemic [7,15,45]. In addition, study findings on the stress perception and related mental health outcomes of healthcare professionals from previous epidemics and pandemics support the findings so far during the COVID-19 pandemic [14,46]. In summary, the literature suggests that nurses are exposed to increased stress both professionally and personally during the COVID-19 pandemic. In addition to a variety of factors, increased pandemic-related concerns and worries, as well as an inadequate supply of pandemic-related information, appear to have a negative impact on the stress experience of nurses. 

However, most of the available studies dealing with the stress experience of nurses during epidemic or pandemic states refer to the inpatient setting or do not consider outpatient care separately, but together with the partial or full inpatient care setting. Indeed, there are a few studies that have qualitatively examined the overall situation in German outpatient care during the COVID-19 pandemic [12,42]. However, the impact of the COVID-19 pandemic on the stress experience and mental health of German outpatient nurses has not yet been adequately clarified. There are no studies that have explicitly analysed the pandemic-related stress experience of German outpatient nurses using a quantitative approach. The influence of pandemic-related concerns on the stress experience of German outpatient nurses has also not yet been investigated. The correlations between pandemic-related stress perception and outpatient nurses’ quality of sleep or work engagement are also still unknown. 

Therefore, understanding pandemic-related stress perception and its impact on the mental health of outpatient nurses is crucial to provide a starting point for preventive measures and improvements that could positively influence the mental health status as well as the career retention of German outpatient nurses during the COVID-19 pandemic. 

### 1.3. Study Aim

This study aims to investigate and describe the stress perception, sleep quality, pandemic-related worries and concerns, as well as the work engagement of outpatient nurses during the COVID-19 pandemic. 

For this purpose, the following assumptions were defined:

**Hypothesis** **1:** *Pandemic-related stress is negatively associated with lower sleep quality among outpatient nurses.*

**Hypothesis** **2:** *Pandemic-related stress is negatively related to lower work engagement among outpatient nurses.*

**Hypothesis** **3:** *Pandemic-related concerns and worries are positively related to higher stress experience among outpatient nurses*.

## 2. Materials and Methods

### 2.1. Study Design and Recruitment 

The present study is a quantitative investigation in the form of a cross-sectional study. For this purpose, an online questionnaire study was conducted among German outpatient nurses from outpatient care services. 

We aimed to include 199 outpatient nurses from Germany in the survey, based on a sample size calculation performed with G*Power (α = 0.05, β = 0.20, d = 0.20) [47].

Recruitment was carried out using multiple channels. A total of 367 outpatient care facilities were contacted by telephone and via email distribution lists of the Federal Association of Private Providers of Social Services. A total of 253 out patientcare services agreed to hand out the flyer about the study to their employees. In comparison, 114 outpatient care services declined participation. Moreover, calls for participation were posted on social networks (Facebook, Xing) to directly address outpatient nurses. Inclusion criteria for recruitment were defined as (1) outpatient nurses, (2) with at least six months of professional experience in outpatient care, and (3) a workload of at least 25 h per week. The online software “Unipark” was used to conduct the survey. 

A cumulative number of 607 prospective participants accessed the online questionnaire between May 2020 and May 2021, of which *n* = 171 (28.2%) initiated and successfully completed the questionnaire, *n* = 315 (51.9%) dropped out, and *n* = 121 (19.9%) did not participate in the survey. 

### 2.2. Measurement and Variables 

The questionnaire used was developed based on a previous literature review. Thus, adaptations of validated and well-established instruments such as the Perceived Stress Scale (PSS) [48,49] and the Pittsburgh Sleep Quality Index (PSQI) [50,51] were used within the survey. In addition, questionnaires used in previous pandemics were reviewed for their applicability in the context of the present study. For example, a questionnaire developed during the A/H1N1 pandemic was incorporated into the survey to measure pandemic-related concerns by outpatient nurses during the COVID-19 pandemic [34]. Furthermore, three items from the Copenhagen Psychosocial Questionnaire (COPSOQ) [52,53] were selected to investigate outpatient nurses’ work engagement during the pandemic. Finally, individually developed items were used to capture socio-demographic and workplace-related characteristics.

#### 2.2.1. Sociodemographic and Workplace-Related Variables 

To assess participants’ characteristics, socio-demographic data was collected using self-constructed items including gender, age, family status, number of children, and level of education. Furthermore, self-constructed items were used to assess workplace-related variables such as information on the work situation e.g., type of employment, shift work, managerial position, length of work experience, as well as the operation area in outpatient care.

#### 2.2.2. Pandemic-Related Stress Perception 

The Perceived Stress Scale (PSS) is a well-known and well-established self-report scale to assess perceived stress [48]. Therefore, the German version of the 10-item Perceived Stress Scale (PSS-10) was used to measure participants’ perceived pandemic-related stress. For this purpose, the original items by Cohen et al. were translated from English into German. All items related to participants’ perceived stress experiences in the last month (e.g., Item 3: How often have felt being nervous or stressed in the past month?) using a five-point Likert scale (0 = never, 1 = seldom, 2 = sometimes, 3 = fairly often, 4 = very often). Since items 4, 5, 7 and 8 were formulated positively, it was necessary to reverse their scores. The PSS-10 total score was calculated by summing up all values of the 10 items and could thus range from 0 to 40. No cut-off scores were set since the PSS is not defined to be a diagnostic tool. However, higher total PSS-10- scores indicated a higher level of perceived stress. Validation of the German version of the PSS-10 proved the instrument to be sufficiently reliable (comparative-fit-index (CFI) = 0.96; Tucker–Lewis Index (TLI) = 0.95; root mean square error of approximation (RMSEA) = 0.07) and highly internally consistent (Cronbach’s α = 0.84) [49]. Moreover, outpatient nurses were asked to rate whether they perceived their lives to have become more stressful since the beginning of the COVID-19 pandemic using a self-constructed item with a five-point Likert scale (1 = I strongly disagree, 2 = I rather disagree, 3 = I neither agree nor disagree, 4 = I rather agree, 5 = I strongly agree).

#### 2.2.3. Sleep Quality 

Two items were used to examine participants’ sleep quality during the COVID-19 pandemic. Firstly, respondents were asked to rate their sleep quality since the beginning of the COVID-19 pandemic (1 = much worse, 2 = worse, 3 = neither worse nor better, 4 = better, 5 = much better). Afterwards, participants were asked about their sleep quality over the past four weeks (“How would you rate the quality of your sleep over the past four weeks?”) on a four-point Likert Scale ranging from “1 = very poor” to “4 = very good”. The latter item was based on an item of the Pittsburgh Sleep Quality Index (PSQI), which is a well-established instrument to assess sleep quality in clinical and non-clinical populations. By recoding the response options of the four-point Likert Scale from 0 to 3, a scale of outpatient nurses’ sleep quality could be determined. According to the PSQI, lower scores are to be interpreted as an indication of healthier sleep quality [50,51]. 

#### 2.2.4. Work Engagement 

To investigate outpatient nurses’ work engagement during the COVID-19 pandemic, three items from the Copenhagen Psychosocial Questionnaire (COPSOQ) 2018 were chosen [52,53]. The COPSOQ is a comprehensive, scientifically validated questionnaire for the assessment of psychological stress and strain at work [54]. On a five-point Likert scale ranging from 1 = never to 5 = always, participants were asked to rate on the following questions (1- vigour) I am full of energy in my work (2- dedication) I am enthusiastic about my work (3- absorption) I am completely absorbed in my work. For further analysis, given answers were recoded to point values (0 = never, 25 = seldom, 50 = sometimes, 75 = fairly often, 100 = always). The sum of the three items was used to calculate the arithmetic scale mean, which reflected the overall level of work engagement among outpatient nurses.

#### 2.2.5. Pandemic-Related Concerns and Worries 

Goulia et al. have developed a questionnaire to assess the pandemic-related concerns and needs of hospital staff during the A/H1N1 pandemic. To date, the authors have not provided information upon validation of the questionnaire [34]. Although the questionnaire was developed during another pandemic, it was deemed suitable for the present study under COVID-19 conditions. Therefore, the questionnaire by Goulia et al. was adapted and translated into German to investigate the pandemic-related concerns and worries of outpatient nurses during the COVID-19 pandemic. At the beginning, a dichotomous question (yes or no) was asked, concerning whether or not the participants were worried about the COVID-19 pandemic. Outpatient nurses were then asked about their perceived general level of worry and concern (1: I worry a little to 9: I worry a lot). This was followed by five questions in a dichotomous format (yes or no) on the issues that the outpatient nurses were most concerned about (danger of the disease, risk of infecting family and relatives, social isolation, and impact of the pandemic on their performance in everyday life). Finally, five items on a nine-point Likert scale followed to explore the perceived level of pandemic-related worries and concerns of the outpatient care workers about specific issues. The items included questions on concerns about the risk of infection (1: very low 9: very high), concerns about serious health consequences of infection, concerns about treatment of infection, perceived employer preparedness for the pandemic, and the need for psychological support regarding pandemic-related worries and fears of outpatient care workers (1: I do not agree at all to 9: I fully agree). The six Likert scale scores for each item were then summed to obtain the arithmetic mean to measure the overall level of pandemic-related worries and concerns of outpatient nurses.

### 2.3. Statistical Methods 

The data were analysed using IBM^®^ SPSS^®^ Statistics (version 27). At first, the data were checked for missing values, statistical outliers (a data point that differs significantly from other values) and plausibility. From the Likert Scales, scale scores were calculated for perceived stress, sleep quality, work engagement, and pandemic-related concerns and worries. For this purpose, recoding of the items was partly necessary. Afterwards, the response patterns within the items and scale scores were descriptively analysed. Scales’ reliabilities were tested by measuring the internal consistencies of the items used for scale calculation with Cronbach’s α (>0.9 excellent, >0.8 good, >0.7 acceptable, >0.6 questionable, ≤0.5 unacceptable) [55]. 

Furthermore, tests for normality were used to determine if the data set was well-modeled by a normal distribution and to decide whether to use parametric or non-parametric tests for further statistical analysis. According to the normality test results, no scales were normally distributed according to Kolmogorov–Smirnov and Shapiro–Wilk Tests (*p* < 0.05). Therefore, non-parametric alternatives were used within the further analyses. 

Moreover, results of the Dunn–Bonferroni tests were considered and the effect sizes of pairwise comparisons were calculated according to r < 0.1 weak effect size, r < 0.3 mediocre effect size and r > 0.5 strong effect size [56]. The statistical level of significance was set at *p* < 0.5. 

To test hypotheses 1 to 3, one-tailed bivariate correlation analyses were conducted to identify correlations between perceived stress, sleep quality, work engagement, and pandemic-related worries and concerns. To determine the correct statistical test for correlation, the necessary criteria of the Spearman’s rank correlation coefficient and Pearson product-moment correlations were checked [57]. The metric data level of the scales was given. In addition, there were no extreme statistical outliers within the data. However, the dependencies between the scales could in part only be described as weakly linear. Furthermore, a normal distribution could only have been assumed regarding the central limit theorem by *n* ≥ 30 [58]. Therefore, Spearman’s rank correlation coefficient was considered more reliable for assessing correlations than the Pearson product-moment correlation. The effects of correlations were interpreted according to r < 0.1 weak effect, r < 0.3 mediocre effect and r > 0.5 strong effect [56]. All *p*-values given were one-tailed (*p* < 0.01), as one direction of effects was assumed for the hypotheses. Taking into account that no statements about directionality or causal relationships between the variables could be made using correlation analysis only [59], additional bivariate linear regression analyses were carried out [57]. For this purpose, the prerequisites of the Gauss–Markov theorem were considered [60]. Random sampling, interval scaling of the independent and dependent variables, linear dependence of the variables and their regression coefficients, exclusion of multicollinearity, given exogeneity, as well as homoscedasticity of the residuals could be established as fulfilled prerequisites. Considering hypothesis 1 and 2, however, the residuals were not normally distributed, which is why additional bootstrapping was applied based on 1000 samples. On the other hand, the residuals of hypothesis 3 were normally distributed, which is why bootstrapping was not used. Effect sizes were interpreted using the standardised beta coefficients with β = 0.1 weak, β = 0.3 moderate, β = 0.5 strong association.

### 2.4. Ethical Considerations 

Ethical approval to conduct this study was obtained from the local psychological ethics committee of the Hamburg Psychosocial Medical of the University Medical Center Hamburg-Eppendorf (UKE) (ethics code: LPEK-0083). Before participating in the study and answering the questionnaire, participants were carefully informed about the purpose, confidentiality of information, anonymity, and voluntariness of the study. Informed consent was then obtained from all participants.

## 3. Results

### 3.1. Sociodemographic and Workplace-Related Variables 

In total, *n* = 171 outpatient nurses answered the questionnaire. However, *n* = 5 participants had not answered the questions on pandemic-related worries and concerns and were therefore excluded from further analysis for better comparability. Thus, an *n* = 166 was considered as the baseline for the data analysis. 

Table 1 depicts the sociodemographic characteristics of the sample. From *n* = 166, 108 were female (65.9%), 56 male (34.1%) and 2 (1.2%) of diverse gender. The majority of participants were between 30 and 39 years old (*n* = 53 or 31.9%), followed by 50 to 59-years old (*n* = 44 or 26.5%), 40 to 49-year olds (*n* = 40 or 24.1%), 18 to 29-year olds (*n* = 17 or 10.2%) and participants over 60 years old (*n* = 12 or 7.2%). Regarding their educational background, more than half of the participants reported having completed intermediate secondary school (*n* = 84 or 50.6%). Furthermore, 43 participants (25.9%) had completed grammar school, 21 (12.7%) specialised grammar school and 18 (10.8%) general secondary school. A total of 92 participants were married (55.4%), whereas nearly a third was unmarried (*n* = 55 or 33.1%). In addition, 10 participants (6.1%) were divorced, eight (4.8%) were in a registered civil partnership and one participant was widowed (0.6%). Most of the participants reported having no children (*n* = 89 or 53.6%), around a third (*n* = 58 or 34.9%) to have one child, 8.4% (*n* = 14) to have two children and 3% (*n* = 5) to have three children.

A large proportion of the respondents had been working in outpatient care for less than five years (*n* = 47 or 28.3%) or six to ten years (*n* = 40 or 24.1%). Furthermore, 24 participants (14.5%) had been working in outpatient care for 11 to 15 years, 27 (16.3%) for 16–20 years and 28 (16.9%) for 21 years and longer. A majority of the participants had a permanent employment contract (*n* = 150 or 90.4%). The distribution of full-time (47%) and part-time work (53%) was almost balanced in the sample. Likewise, the distribution of shift work was balanced. Around 52.4% of the respondents stated that they worked in shifts, while 47.6% stated they did not. In addition, 36.1% (*n* = 60) of the respondents in the sample reported working in managerial positions. Furthermore, 3% (*n* = 5) provided outpatient care in nursing homes and 2.4% (*n* = 4) in intensive care. The remaining 14 participants (8.4%) belonged to other, unspecified areas of operation in outpatient care.

### 3.2. Pandemic-Related Stress Perception 

Response patterns revealed that 27.7% (*n* = 46) of the participants rather agreed and 22.9% (*n* = 38) fully agreed that their lives had become more stressful since the onset of the COVID-19 pandemic. On the other hand, 24.1% (*n* = 40) neither agreed nor disagreed, 18.1% (*n* = 30) of the participants rather disagreed and 7.2% (*n* = 12) strongly disagreed that they felt that their lives had become more stressful since the beginning of the COVID-19 pandemic. 

The calculation of the PSS-10 scores showed a minimum of seven and a maximum of 32, with M (SD) = 18.83 (±5.48), a mode of 19 (*n* = 18) and a median of 18.5. There were some outliers with very high PSS-10 scores, which were considered individually and included as plausible, as they also reported poorer sleep quality and lower work engagement. It could be demonstrated that half of the participants had rather low stress levels, corresponding to a PSS-10 score of 18.5 or less. In addition, two-thirds of the participants had a PSS-10 score of up to a maximum of 20, which is considered a moderate stress level. However, the remaining third scored from 21 to a maximum of 32 on the scale, indicating a rather elevated stress experience during the COVID-19 pandemic.

### 3.3. Sleep Quality 

Sleep quality of the outpatient nurses interviewed was analysed descriptively. Thirty-one participants (18.7%) perceived their sleep patterns to have become much worse and 47 (28.3%) worse since the beginning of the COVID-19 pandemic. However, more than half of the participants (*n* = 87 or 52.4%) did not notice any difference in their sleeping patterns and one participant (0.6%) even perceived much better sleep patterns. Regarding their perceived sleep quality over the past four weeks, 97 (58.4%) participants reported having a good and 6 (3.6%) to have a very good quality of sleep, whereas 59 (35.5%) participants perceived their quality of sleep as poor and 4 (2.4%) very poor.

The PSQI scale had a minimum score of 0 and a maximum of 3, with M (SD) = 1.37 (±0.6), a mode of 1 (*n* = 97) and a median of 1. Since a majority of the respondents (*n* = 103 or 62%) scored 0 and 1 on the PSQI scale, it can be stated that a majority of respondents can be considered to have had good to very good sleep quality. On the other hand, 63 (37.9%) of the outpatient nurses had scores of 2 and 3, indicating poor or very poor sleep quality since the onset of the COVID-19 pandemic. Overall, then, the descriptive analysis indicates that a majority of participants had good sleep quality during the COVID-19 pandemic.

### 3.4. Work Engagement 

A descriptive analysis of the items on work engagement was conducted. The analysis revealed that a majority (*n* = 79, 47.6%) stated that they were fairly often, and 30 participants (18.1%) stated that they were always, full of energy at work. On the other hand, 33 (19.9%) stated they were sometimes, 21 (12.7%) seldom and three (1.8%) stated they were never full of energy at work. Considering their dedication at work, 67 (40.4%) participants perceived themselves to be fairly often, and 29 (17.5%) to be always enthusiastic about their work. However, 49 participants (29.5%) were sometimes, 18 (10.8%) seldom and three (1.8%) were never enthusiastic about their work. Additionally, 61 (36.7%) outpatient nurses had the feeling of being sometimes, 55 (33.1%) to be fairly often and 15 (9%) to be always fully absorbed in their work, whereas 27 (16.3%) stated that they were seldom and 8 (4.8%) never perceived full absorption in their work. 

The calculation of the scale scores revealed that the minimum of overall work engagement achieved was 0 and the maximum was 100. Furthermore, the mean was identified as M (SD) = 62.8 (±20.85), the mode as 75 (*n* = 34) and the median as 66.67. A majority of participants’ work engagement during the COVID-19 pandemic was characterised by high vigour, high dedication, and moderate absorption. Overall work engagement can thus be described as tending to be high. 

### 3.5. Pandemic-Related Worries and Concerns 

To the first dichotomous question, 57 (34.3%) denied and 109 (65.7%) confirmed that they were worried about the COVID-19 pandemic. The response distributions to the dichotomous questions showed what outpatient nurses were mostly worried about. Most respondents were concerned about the risk of infection to their family and relatives (*n* = 117 or 70.5%), isolation from their family and social environment (*n* = 103 or 62%), as well as the risk of infection associated with their job (*n* = 109 or 65.7%). Response patterns to the question about the risk of the disease and the impact of the pandemic on outpatient nurses’ performance were more evenly distributed. A total of 53.6% (*n* = 89) of participants stated they were mostly concerned about the risk posed by COVID-19 and 44.6% (*n* = 74) were concerned about the influence of the pandemic on their daily performance. For the items with 9-point Likert scales, 41 outpatient nurses (24.7%) worried very little (1: I worry very little, 9: I worry a lot) about the COVID-19 pandemic. Overall, the median for the degree of worry was rather low with 4/9 on the Likert scale. Thus, only 26 (15.7%) participants were very concerned about the COVID-19 pandemic, consequently indicating their level of concern in the range of 7 to 9. Furthermore, the majority of outpatient nurses rated their occupational risk of contracting COVID-19 (1: very low, 9: very high) in the middle of the Likert scale (*n* = 28 or 16.9%), with response options 6 (*n* = 24 or 14.5%) and 7 (*n* = 25 or 15.1%) also being mentioned with comparatively equal frequency. Nevertheless, the median perceived risk of contracting COVID-19 was moderate at 5/9. Interestingly, around 60% (*n* = 99 or 59.8%) of outpatient nurses were relatively unconcerned about the health consequences of being infected with COVID-19, indicating their level of concern as between 1 to 4 on the Likert scale. On the other hand, a minority of 16 participants (9.6%) were very concerned about the health consequences of infection with the virus, giving a score of 9 on the Likert scale. The median level of concern about health consequences in the event of infection was low at 4/9. Besides, worries about an easy treatment of infection peaked in the middle of the Likert scale (*n* = 40 or 24.1%), while 11 participants (6.6%) strongly disagreed with the statement that they were very concerned about treatment for COVID-19. In this regard, the median score of 5/9 on the Likert scale indicated a moderate level of concern among participants. Response patterns to the question about their employer’s perceived preparedness for the challenges of the pandemic showed that 67 (40.4%) participants tended to agree, scoring 1 to 4 on the Likert scale, and 76 (45.7%) tended to disagree that they felt their employer was well prepared for the pandemic. The median reached up to 5/9, indicating moderate concern among ambulatory care workers about their staff members’ perceived readiness to face the challenges of the pandemic. The majority of outpatient nurses disagreed that they needed pandemic-related psychological counselling to address the challenges of the pandemic by selecting 1 to 4 on the Likert scale (*n* = 95 or 57.3%). However, a minority of 11 participants (6.6%) fully agreed that psychological support was necessary for them. Still, the majority of participants did not tend to see the need for a supportive psychological service for the challenges of the COVID-19 pandemic (median: 4/9). 

Calculating the total score for pandemic-related concerns and worries resulted in a minimum of 1 and a maximum of 7. The mean was M (SD) = 4.5 (±1.46), the median 4.5 and the mode 4.2. 

### 3.6. Correlation Analysis 

Spearman rank correlations were calculated for perceived stress, sleep quality, work engagement, pandemic-related concerns and worries (see Table 2). 

A significantly weak positive correlation was found between PSS-10 and PSQI (r = 0.264, *p* ≤ 0.001). Thus, there is an indication that pandemic-related stress is related to the sleep quality of outpatient nurses. Hypothesis 1 can be confirmed. Regarding hypothesis 2, a significantly weak negative correlation was found between PSS-10 and the work engagement of outpatient nurses (r = −0.223, *p* = 0.002). Thus, pandemic-related stress also influenced the work engagement of outpatient nurses within our study. However, no significance was found for the correlations between PSS-10 and pandemic-related concerns and worries (r = 0.039, *p* = 0.307). The non-significant correlations thus indicate that hypothesis 3 must be rejected. However, these initial indications are to be further specified with the help of regression modelling with regard to the direction of the effects and the effect sizes.

### 3.7. Regression Modelling 

Regression modelling with bootstrapping performed on 1000 samples with sleep quality as the criterion and perceived stress as the predictor was significant (R^2^ = 0.073; F (1, 164) = 12.883, *p* < 0.001). The regression coefficient of the variable PSS-10 was 0.029 and was significant (t (164) = 3.589; *p* < 0.001). Thus, 7.3% of the variance of sleep quality can be explained by the variable perceived stress. Consequently, perceived stress is a significant predictor of sleep quality, as the sleep quality scale score increases by 0.029 for each additional point on the perceived stress scale. The effect size proved to be moderate with (β = 0.270). Thus, it was demonstrated that pandemic-related stress decreased the quality of sleep of outpatient nurses within our study, and so hypothesis 1 can be confirmed. Similarly, regression modelling with bootstrapping was performed on 1000 samples with work engagement as a constant and perceived stress (PSS-10) as a predictor, which turned out to be significant (R^2^ = 0.053; F (1, 164) = 9.196, *p* = 0.003). Accordingly, 5.3% of the variance in work engagement can be explained by the variable PSS-10. The regression coefficient of the variable perceived stress is -0.878 and significant (t (164) = −3.033; *p* < 0.001). Perceived stress thus proves to be a significant predictor of work engagement among outpatient nurses, as work engagement decreases by 0.878 points for each additional point on the PSS-10 scale. According to the beta-coefficients, this corresponds to small or moderate effect with β = −0.230. Thus, hypothesis 2 can also be confirmed, as pandemic-related stress is a negative predictor for the work engagement of outpatient nurses. The attempt to model a linear regression with PSS-10 as constant and pandemic-related worries and concerns as a predictor failed in terms of significance (R^2^ = 0.024; F (1, 164) = 0. 093, *p* = 0.760). In summary, hypothesis 3 must therefore be rejected, as pandemic-related worries and concerns were not suitable for predicting perceived stress in the present study (see Table 3).

In summary, the following conclusions can be drawn regarding the hypotheses: 

Hypothesis 1 was accepted. Pandemic-related stress proved to be a predictor of poorer quality of sleep among outpatient nurses. The effect size proved to be moderate. 

Hypothesis 2 was accepted. Pandemic-related stress proved to be a predictor of lower work engagement among outpatient nurses. The effect size was moderate. 

Hypothesis 3 was rejected. Pandemic-related concerns and worries were not positively related to higher stress experience among outpatient nurses.

## 4. Discussion

To our knowledge, this is one of the first exploratory quantitative studies to examine the stress experience of German outpatient nurses during the COVID-19 pandemic and associations related to their sleep quality, work engagement, and pandemic-related worries and concerns. Overall, the outpatient nurses in our study were found to have rather moderate pandemic-related stress levels. In addition, outpatient nurses reported rather good sleep quality, as well as moderate to high work engagement and moderate pandemic-related worries and concerns. Concerning assumptions 1 and 2, it was shown that pandemic-related stress was a predictor of lower sleep quality and work engagement among outpatient nurses. However, no association could be found between pandemic-related stress and pandemic-related worries. 

### 4.1. Descriptive Analysis of Pandemic-Related Stress Perception, Sleep Quality, Work Engagement and Concerns 

More than half of the participants perceived their lives to be more stressful since the beginning of the COVID-19 pandemic. In addition, about one third of respondents were found to have comparatively higher stress levels, which is consistent with the findings of other studies on the COVID-19 pandemic as well as on previous epidemics [4,5,14,37,42,61]. Unexpectedly, however, a majority of the outpatient nurses in the present study reported only a mild to moderate level of stress. This result contrasts with the findings of the studies mentioned above, in which the vast majority of nurses reported significantly increased epidemic or pandemic-related stress levels. Moreover, the stressful working conditions in outpatient care prior to the COVID-19 pandemic suggested that summing the old and new pandemic-related stressors would have resulted in consistently significantly higher stress levels in the study group. The question therefore arises as to what causes outpatient nurses to be less affected by pandemic-related stress. One possible explanation could be the lower contact with COVID-19-positive patients in outpatient care compared to inpatient or intensive care settings. For example, many studies have shown that stress levels and psychological distress are higher among nurses who are in direct contact with COVID-19 patients than among those without contact with COVID-19 patients [5,13,17,37,61]. In addition, nurses’ strong sense of responsibility and professional commitment are discussed as possible stress-buffering reasons for lower pandemic-related stress levels [62]. The fact that nurses show a strong sense of responsibility, commitment to their profession, and concern for the well-being of their patients has also been found in studies from the German context [3,9,32]. It could therefore be assumed that a strong professional commitment buffered the pandemic-related stress experience of outpatient nurses in this study as well. Following the transactional stress model [63,64], it can also be assumed that the risk from the stressors of the pandemic could be assessed as moderate to low by the outpatient nurses. At the same time, it can be assumed that they positively assessed their coping resources to deal with the pandemic-related stressors, which led to a moderate stress level overall. However, it remains unknown what exact resources were available to the outpatient nurses and what coping strategies they used. Returning to the JD-R model [65], resources such as autonomy from working alone [66], increased resilience from increased social recognition since the onset of the COVID-19 pandemic [62], and the high level of pandemic-related information in the present sample could be some of the possible resources that buffer the stress experience of outpatient nurses [34,36]. However, all these considerations remain hypotheses that need to be tested. 

Half of the outpatient nurses reported deterioration in their sleep quality since the onset of the COVID-19 pandemic, while the other half did not notice any change in their sleep patterns. However, only about one third of the participants also reported poor or very poor sleep quality and two-thirds even reported good or very good sleep quality. The calculation of the PSQI scores also showed an average good sleep quality of the participants during the COVID-19 pandemic. These results are indeed surprising and latently contradictory compared to the predominant consensus in the literature. A large number of studies show that the sleep quality of nurses decreased during the COVID-19 pandemic, which was mainly characterised by sleep disturbances and insomnia [7,15,24]. Comparable findings were also found in previous pandemics such as SARS, MERS or Ebola [14,25,62,67]. However, all of these studies were predominately related to inpatient care. It should also be noted that the baseline sleep quality of the participants in the past is unknown, making an accurate comparison difficult. In addition, the factor of proximity to COVID-19 positive patients might also play a role in nurses sleep quality, as closer proximity to COVID-19 positive patients seemed to negatively influence the sleep quality of nurses [7,14,25,61,68]. Consequently, it would be a logical conclusion that outpatient nurses did not rate their sleep quality too poorly, even though other results would have been expected. 

A high level of work engagement was found among the majority of the outpatient nurses. The fact that nurses’ work engagement is high despite all the challenges of the pandemic has also been found in studies from the national and international context [13,62,69]. In this context, Giménez-Espert et al. concluded that nurses’ work engagement may be even higher than ever before due to the extreme circumstances of the COVID-19 pandemic, which could be due to their high resilience and awareness of the enormous importance of their work [62]. This statement is also consistent with the findings of Wildgruber et al., who found that nurses’ work engagement was increased during the COVID-19 pandemic due to heightened concerns about the well-being of their patients [13]. However, the extent to which resilience of the outpatient nurses or concern for the well-being of their clients is pronounced among the outpatient nurses in the present study cannot be determined at this point, which would need to be verified.

In addition, research findings revealed that outpatient nurses within the present study were mostly worried about the risk of COVID-19 for their relatives and their families, their occupational increased risk of infection, and isolation from their families and social environment. Moreover, outpatient nurses had pronounced concerns about the treatment of COVID-19, compared to the threat to their health posed by the disease. On the other hand, outpatient nurses perceived few worries about possible influences of the pandemic on their daily performance. In addition, the majority of nurses felt that they did not need psychological support for the challenges of the pandemic. Opinions on the employer’s preparedness for the challenges of the pandemic were differentiated. The median scores of the items and the calculation of the total score of pandemic-related worries and concerns indicate that the outpatient care workers were only moderately concerned about the COVID-19 pandemic. Our findings are consistent with those of the study from Sahashi et al. in which healthcare professionals were most concerned about their own risk of infection or the infection of their family and friends. However, the overall level of pandemic-related concerns and worries in this study was significantly higher than the overall level of concern among outpatient nurses in our study [34]. Goulia et al. found comparable results on key pandemic-related concerns and worries among health professionals during the A/H1N1 influenza pandemic, where more than half of the participants were concerned about the pandemic and worried about the risk of infection for family and friends. As in this study, levels of concern, perceived risk of contracting A/H1N1, perceived health consequences if infected, perceived employer preparedness, and concern that the infection would be difficult to treat were only moderately high. However, the existence of psychological support for concerns about the A/H1N1 influenza was considered more important than among outpatient nurses in this study [34]. It is questionable why pandemic-related concerns were unexpectedly moderate among outpatient nurses. It could be argued that fewer direct contacts with COVID-19 patients may have positively influenced their level of concern. However, in the study by Sahashi et al., no difference was observed between the physical proximity to COVID-19 patients and the level of worries among healthcare professionals [36]. It is possible that other factors played a buffering role in the pandemic-related concerns and fears of outpatient nurses, which were not captured in this study.

### 4.2. Associations between Pandemic-Related Stress and Sleep Quality 

Hypothesis 1 could be confirmed insofar as the participants in our study tended to show decreased sleep quality with increasing stress levels. It should be noted, however, that the effect size of the association between pandemic-related stress and sleep quality was rather moderate. This result is noteworthy in that evidence in the literature would suggest a much stronger relationship between the two variables. During the SARS outbreak in 2003, Maunder et al. found that high stress in the work setting and a deterioration in sleep quality were among the first effects of a pandemic outbreak on healthcare professionals [41]. Increased workload increases perceived stress, which in turn can affect sleep quality and trigger sleep disorders. It was commonly known before the onset of the COVID-19 pandemic that the workload in outpatient care was high and steadily increasing [19]. For example, even before the outbreak of the COVID-19 pandemic, Kunzweiler et al. found a correlation between the increase in workload and sleep disorders among nurses [23]. As the COVID-19 pandemic progressed, the literature pointed out that workload in ambulatory care increased even more, e.g., due to staff absences or increased hygiene and safety measures to prevent infection [10]. In addition, increased reports of primary and secondary stressor appraisals predict poorer sleep quality and lower psychological well-being, and vice versa [67]. It was therefore expected that the stress level of outpatient nurses would have had a much greater impact on sleep quality. However, the pandemic-related stress level of our participants was rather moderate, which could explain to some extent that their stress had less impact on their sleep quality. It is also conceivable that a habituation or adaptation to pandemic-related stressors occurred over time, so that outpatient nurses perceived the threat of pandemic-related stressors as less risky. In fact, such a gradual psychological adjustment to pandemic-related stressors, leading to a decrease in insomnia, was already observed among health professionals during the SARS pandemic [25]. It should be noted, however, that these explanations are purely speculative, as we were not able to establish causal relationships or analyse temporal differences or the influence of resources on sleep quality among outpatient nurses in our study. 

### 4.3. Associations between Pandemic-Related Stress and Work Engagement 

Hypothesis 2 could be confirmed, as our study participants showed decreasing work engagement with increasing pandemic-related stress. Our study results are thus in line with the German study by Wildgruber et al. who found that the work engagement of nurses decreases with increasing pandemic-related stress [13]. In addition, there is overlap with international study findings by Allande-Cussó et al. [69] who identified high work engagement among Spanish nurses, as well as with the study by Zhang et al. [44], who found low stress and high work engagement among Chinese nurses during the COVID-19 pandemic. The latter study also found a negative correlation between pandemic-related stress and work engagement. Furthermore, in the Spanish study by Giménez-Espert et al. (2020), work engagement of nurses proved to be high despite high stress and low available resources [62]. Thus, it seems that there are other factors that can explain the relationship between work engagement and pandemic-related stress. Indeed, studies have shown that personal resources such as high resilience can increase work engagement [70]. High levels of resilience may result from nurses’ awareness that their work is more important than ever in maintaining patient care during these times [62]. In addition, Zhang et al. discussed personal resources, family status and support, organisational factors, and even socioeconomic factors as further determinants of work engagement [44]. However, the aim of hypothesis 2 was to model the impact of pandemic-related stress on outpatient nurses’ work engagement, not to accurately predict their work engagement during the COVID-19 pandemic [13]. Thus, the actual aim of proving a relationship between stress and work engagement can be considered fulfilled, but this offers further potential for further studies. 

### 4.4. Associations between Pandemic-Related Concerns and Pandemic-Related Stress

The fact that we could not confirm any associations between pandemic-related concerns and pandemic-related stress in our study (Hypothesis 3) is rather striking. Already during the A/H1N1 pandemic, an independent association between increased worry and increased stress among healthcare professionals was found. In particular, concerns about self-infection or infection of family and friends triggered stress among the study participants [34]. These concerns were also among the main concerns of our study participants. In addition, worries about family and friends, temporary lack of protective equipment, fear of the consequences and treatment of an infection, as well as worries due to the increased occupational risk of infection were also identified as causing stress among health care professionals during the COVID-19, regardless of the setting [8,35,36]. Similarly, outpatient nurses in our study were primarily concerned about the well-being of their family and friends, isolation from their family and friend and their increased occupational risk of infection. However, their level of concern proved to be moderate and no association with their experience of stress was found, which is concerning. As the scope of questions related to pandemic concern was limited in our study, more precise explanations of this remarkable result are not possible. There is evidence that resilience [71,72] can mediate pandemic-related concerns. However, our data also do not allow us to draw conclusions about the causes or determinants of the rather moderate level of worry and the lack of association with perceived stress.

### 4.5. Strengths and Limitations 

The present study has provided important insights into the stress experience of German outpatient nurses during the COVID-19 pandemic. The results were partly surprising and contradict the prevailing knowledge in the literature, which predominantly points to high stress levels among nurses during the COVID-19 pandemic. Therefore, this study highlights the complexity of the human psyche in relation to the subjective experience of stress. A strength of the present study is the fact that the sample had a high degree of differentiation in terms of age distribution, work experience and areas of operation in outpatient care that could be analysed. This allowed a wide range of perspectives of outpatient nurses regarding their stress experience during the COVID-19 pandemic to be collected. Furthermore, most of the items and thus the scales (e.g., PSS-10, COPSOQ, PSQI) were developed on the basis of well-established, validated, and highly internally consistent instruments [49,51,54]. Consequently, the reliability of the scales in the present study could also be confirmed with good to very good internal consistencies according to Cronbach’s α; with the exception of the PSQI, as it was a single-item scale. All other scales consisted of at least three up to ten items. In addition, for pandemic-related concerns and worries, a questionnaire was used that had proven to be a useful survey instrument during the A/H1N1 pandemic [34]. For example, Sahashi et al. also used the questionnaire in their study to assess pandemic-related concerns and the level of information among healthcare professionals during the COVID-19 pandemic [36]. The section with questions on sleep quality was the shortest, with a total of two items. In hindsight, it would have been useful to include more questions on the exact characteristics and perceived causes of the deterioration in sleep quality to better relate the responses to pandemic-related causes. 

A limitation of the study is the limited representativeness of the results. Differences that are noticeable between other researchers’ results and our results may be due to the rather small size of the research sample included in this study.

Therefore, only a limited number of care facilities and outpatient nurses could be included in the study. Thus, the total number of evaluated response sets (*n* = 166) was comparatively small compared to the basic population of outpatient nurses in Germany (N = 422,000) [73]. Consequently, it is difficult to draw conclusions about the stress experienced during the COVID-19 pandemic for all outpatient nurses in Germany. It is also noticeable that significantly more women participated in the study. Even though it must be emphasised that significantly more women work in the outpatient care sector than men [73], the overrepresentation of the female gender could have affected the results of the study. In addition, the response rate of this study was 28.2%, which is why non-response bias due to systematic failures to respond cannot be excluded [74]. A bias of the results to self-selection cannot be ruled out either, as the outpatient nurses were either too stressed to participate in the study or because they were not stressed at all and therefore had no interest in this survey. 

Furthermore, it must be taken into account that the study is limited due to its cross-sectional study-design. Firstly, the results do not allow for any conclusions to be drawn about causal relationships. Furthermore, the survey data are based exclusively on self-reporting, which can promote response bias [49]. Besides, the cross-sectional design results in a lack of follow-up. Therefore, it would be necessary to investigate the medium- and long-term consequences of the circumstances and possible associations with related mental health outcomes for outpatient nurses during the COVID-19 pandemic. In this context, another limitation of the study is the fact that the survey period included several waves of the COVID-19 pandemic. Habituation effects to the experience of stress that could occur, as well as new coping strategies that outpatient nurses may have developed, could thus not be taken into account. Therefore, it cannot be ruled out that the experience of stress differed between the waves which were not analysed within the present study. In this context, it should also be taken into account that perceived stress is influenced by multifactorial psychosocial aspects of daily life. It is therefore to be expected that the predictive power of the PSS decreases after a few weeks [48]. As the other scales used also interrogated snapshots of multifactorial psychosocial and socially influenceable constructs, the same can be assumed for sleep quality, work engagement, and pandemic-related worries and concerns. The results of the present study can therefore only be regarded as a rough estimate that can only provide indications of psychological distress during the COVID-19 pandemic and the possible development of mental disorders.

Finally, our study of the stress experience of outpatient nurses during the COVID-19 pandemic was limited to a few sub-aspects of stress experience. Thus, it may be that there are other relevant factors influencing participants’ stress experience that were not covered within our questionnaire. Similarly, possible resources, coping mechanisms and protective factors of outpatient nurses that could have a buffering effect on their stress experience were not captured by the questionnaire. 

### 4.6. Implications for Research and Practice

#### 4.6.1. Implications for Research

Since the present study revealed an unexpectedly moderate stress experience of outpatient nurses during the COVID-19 pandemic, future research should focus on identifying reasons for this. Possibly, the individual perspectives of outpatient nurses may need to be brought more into the focus of research. This could be done, for example, with the help of qualitative research methods. Here, individual or group interviews could provide new insights that could better explain or support the present study results. Furthermore, longitudinal studies with larger samples would be needed to investigate causal links between pandemic-related stress experiences and related mental health outcomes, that could not be captured in this study [59]. In this regard, an expansion of the questionnaire with additional items would be worth considering in order to gain even more detailed insights into the stress experience of outpatient nurses. Items for recording stress buffer factors could also be included. In addition, the scales to capture the stress experience, sleep quality and work engagement of the target group could be tailored more specifically to outpatient nurses, as the items and scales used in this study were developed based on the general population. As a less expensive alternative to a longitudinal study, a follow-up cross-sectional study, e.g., after one year, could also be considered for comparisons. Given sufficient time and financial resources, a mix of methods could capture both qualitative and quantitative results to provide a holistic overall picture of outpatient nurses’ stress experience during the COVID-19 pandemic. Finally, workplace health intervention studies would be useful to investigate the impact of behavioural and organisational workplace health promotion interventions on outpatient nurses’ stress experience, related mental health outcomes and their overall well-being. By conducting such evaluation studies in the workplace setting, a continuous improvement process of workplace health promotion measures could be initiated. In this way, the measures implemented could be continuously monitored, adapted and expanded. 

#### 4.6.2. Implications for Practice

In order to reduce and prevent a further increase in pandemic-related stress, the implementation of comprehensive conceptual approaches from behavioural and organisational measures of workplace health promotion in outpatient care services is recommended. In this context, outpatient care services should pay more attention to empowering and enabling their staff to strengthen their personal and work-related resources as well as coping strategies to deal with pandemic-related stress. In this context, employers should ensure that they provide the necessary pandemic-compliant health promotion training to their staff. In addition, it has been shown that the factor of social or collegial support in coping with stress should not be underestimated. However, social exchange among colleagues or with family and friends is more difficult in times of a pandemic, as hygiene and distancing measures take effect. Setting-specific conditions and existing resources must be taken into account [75]. However, the implementation of organisational measures would be preferable to behavioural measures, as they have a wider reach and are less dependent on individual compliance [76]. In addition, there is evidence that nurses may have difficulty implementing behavioural measures due to the high workload and their busy schedules [12]. In any case, employees should be involved in the development of interventions in order to target their needs and to explain the benefits of the interventions to them accordingly. This approach could contribute to the effectiveness of the interventions, which, however, would need to be documented and evaluated after implementation [77]. On the employer side, behavioural health interventions could start on a small scale by ensuring that outpatient nurses can take their breaks [10,12,19,21] and can meet their basic needs despite pandemic-related extra workload [3]. In addition, physical activity and nutrition of outpatient nurses seemed to be impaired in the context of the COVID-19 pandemic, so physical activity and a healthy diet should not be neglected to maintain good health during the pandemic [8]. Moreover, courses on stress management, sleep hygiene, resilience, and relaxation could help outpatient nurses to better cope with stress levels during the pandemic [67,78]. In this context, employers should ensure that they provide the necessary pandemic-compliant health promotion training to their employees. Furthermore, it has been shown that the factor of social or collegial support in coping with stress should not be underestimated. However, social exchange among colleagues or with family and friends is more difficult in times of a pandemic, as hygiene and distancing measures take effect. It should also be taken into account that outpatient nurses usually work alone, so that social support from colleagues or superiors is hardly possible anyway [31,79,80,81,82]. Outpatient care services should therefore actively promote communicative exchange among their staff and supervisors by creating and offering pandemic-compliant opportunities for this, e.g., digital exchange platforms or messenger services. In the private sphere, on the other hand, outpatient nurses should try to obtain sufficient social support from their social environment on their own initiative. In doing so, care should be taken to ensure that private life is maintained despite the social restrictions caused by the pandemic [8,83,84]. Following the JD-R model and the transactional stress model, social support can therefore function both as a job resource and as a coping strategy [43]. 

Furthermore, outpatient nurses showed a relatively high level of work engagement, which was shown to mitigate their PSS-10. Subsequently, work engagement should be further promoted to continue to benefit from this effect. As work engagement increases in resource-rich work environments, outpatient care services should focus more on providing sufficient work resources to their staff. These include, for example, regular feedback, recognition and support, a good leadership style, and the provision of information and training [43]. In the present study, pandemic-related informedness turned out to be a distinctly available resource, as the participants reported to be generally well-informed about COVID-19. Nevertheless, the literature repeatedly points out that there were still deficits in pandemic-related information among healthcare professionals during the COVID-19 pandemic [35,37,42,85]. Therefore, despite the results of our study, it can only be reiterated that the dissemination of regular pandemic-related information and training opportunities remains essential, as this appears to be an important key to supporting healthcare workers in a pandemic situation. As a positive side effect, the study by Goulia et al. also found a correlation between pandemic-related information and reduced pandemic-related worries, which could also be transferred to outpatient nurses [34]. However, employers need to use an appropriate communication strategy to convey the information. In addition, due to the dynamic innovations and changes in the pandemic, employers need to be more flexible in the information and training they provide. Training and information should therefore be regularly updated and adapted [83]. In this context, the study by Hetzmann et al. also pointed out that the level of digitalisation in outpatient care services may still need to be improved in order to ensure adequate information transfer. However, due to the limited financial resources of outpatient care services, this would also require government subsidies [42]. As the general average age in outpatient care is rising [73], attention must also be paid to ensuring that older employees in particular are trained in the use of digitalised tools where appropriate.

Despite moderate levels of stress among two-thirds of the outpatient nurses in the present study, one third reported a tendency towards high levels of stress during the COVID-19 pandemic. In addition, some foci of pandemic-related worry and concern were evident among participants, although the overall level of worry was also moderate. Indeed, a majority of the participants in our study declined to seek emergency psychotherapy services. However, the dynamic developments of the pandemic cannot exclude spontaneous worsening of pandemic-related distress. In addition, the concerns and needs of the more stressed minorities must be taken seriously and perceived, which underlines the importance of a psychological emergency service. Therefore, implementation within outpatient care services would be advisable to create a point of contact that can be used voluntarily in cases of perceived psychological distress and increased pandemic-related concerns during this time [2,83]. It would be advisable to offer this service as low-threshold as possible, i.e., by using digital solutions in the form of telecommunications [86]. A digital offer would also be the preferred choice in the context of infection prevention. In addition, the outsourcing of a psychotherapeutic emergency service could be a quick and cost-effective solution due to the limited human and financial resources of outpatient care services [42]. 

## 5. Conclusions 

The results of this study provide important insights and a deeper understanding of outpatient nurses’ stress perception during the COVID-19 pandemic, but also reveal some surprising findings that contradict previous findings in the literature. 

The present study showed that German outpatient nurses had rather moderate pandemic-related stress levels and little pandemic-related worries during the COVID-19 pandemic, as well as rather good sleep quality and rather high pandemic-related information levels. The results suggest that differentiation of care settings must be important when assessing the stress experience of nurses due to the different baseline conditions in inpatient and outpatient care. There are open questions about the moderate stress level of the outpatient nurses and its causes. The determinants of the missing associations between stress, pandemic-related worries and concerns of outpatient nurses also remain open. Furthermore, it is also questionable what personal and work-related resources were available to outpatient nurses or what coping strategies they used to manage stressors during the COVID-19 pandemic. 

Although our study has so far revealed only moderate stress levels in the participants, a spontaneous deterioration of stress levels due to the dynamic changes of the pandemic cannot be excluded. In addition, it should be emphasised that there were also more stressed minorities among the study participants, whose mental health status must be taken into account and whose needs must be addressed. The creation of health-promoting working conditions through a comprehensive concept of low-threshold behavioural and organisational measures of workplace health promotion could thus contribute to keeping the pandemic-related stress experience of outpatient nurses at a low to medium level. To this end, opportunities should be created for outpatient nurses to promote their resources through the use of work resources and health-promoting offers despite increased workload during the COVID-19 pandemic [10,12,19]. In view of the findings that increased pandemic-related stress has a negative impact on the work engagement of outpatient nurses, aspects to promote work engagement in particular should be taken into account in the development of workplace health promotion measures. Overall, and despite the results of this study, the effects of pandemic-related stress on the mental health of outpatient nurses should by no means be underestimated. Therefore, further in-depth research about the study group and the development of target group-specific measures that take into account the specific conditions in outpatient care are indispensable to contain their stress experience and the development of adverse mental health outcomes during the COVID-19 pandemic.

## Figures and Tables

**Table 1:** Sociodemographic characteristics.

Sociodemographic Characteristics	*n* (%)
*Gender*	
Male	56 (33.7%)
Female	108 (65.1%)
Diverse	2 (1.2%)
*Age*	
18–29 years	17 (10.2%)
30–39 years	53 (31.9%)
40–49 years	40 (24.1%)
50–59 years	44 (26.5%)
≥60 years	12 (7.2%)
*Highest education*	
General secondary school	18 (10.8%)
Intermediate secondary school	84 (50.6%)
Specialized grammar school	21 (12.7%)
Grammar school	43 (25.9%)
*Family status*	
Unmarried	55 (33.1%)
Married	92 (55.4%)
Registered civil partnership	8 (4.8%)
Divorced	10 (6%)
Widowed	1 (0.6%)
*Number of children*	
No children	89 (53.6%)
1 child	58 (34.9%)
2 children	14 (8.4%)
3 children	5 (3%)

**Table 2:** Spearman rank correlations (*n* = 166).

Variables	1	2	3	4	5
Perceived Stress	--				
Work engagement	−0.223 **	--			
Pandemic-related concerns and worries	0.039	−0.014	--		
Sleep quality	0.264 **	−0.204 **	0.200 **	−0.208 *	--

Note: ** Correlation is significant at the 0.01 level (1-tailed). * Correlation is significant at the 0.05 level (1-tailed).

**Table 3:** Linear regression modelling, (*n* = 166).

		Unstandardized Coefficients		95% Confidence Interval		Standardized Coefficients		Regression Results
Hypothesis	Variables	B	SE	Lower	Upper	β	t	
1	(Constant)	0.814 **	0.157	0.512	1.141	-	5.077 **	R = 2.70 R^2^ = 0.073 F = 12.883 *p* < 0.001
	PSS-10	0.029 *	0.008	0.013	0.045	0.270	3.589 **	
2	(Constant)	79.327 **	6.103	67.372	91.784	-	13.981 **	R = 2.30 R^2^ = 0.053 F = 9.196 *p* = 0.003
	PSS-10	−0.878 *	0.326	−1.526	−0.291	−0.230	−3.033 *	
3 ^b^	(Constant)	18.429	1.384	-	-	-	13.316 **	R = 0.024 R^2^ = 0.001 F = 0.093 *p* = 0.760
	Pandemic-related concerns and worries	0.089	0.292	-	-	0.024	0.305	

Note: Unless otherwise noted (b), bootstrap results are based on 1000 bootstrap samples; ** *p* < 0.001, * *p* < 0.01.

## Data Availability

The datasets generated and/or analysed during the current study are not publicly available due to German data protection regulations but are available from the corresponding author on reasonable request.
